# Breast-Conserving Surgery in Multicentric Breast Cancer: Evolving Evidence and Patient Selection

**DOI:** 10.3390/cancers18162705

**Published:** 2026-08-21

**Authors:** Mariam Rizk, Kefah Mokbel

**Affiliations:** 1 The London Breast Institute, Princess Grace Hospital, London W1U 5NY, UK; 2 Burjeel Cancer Institute, Burjeel Medical City, Abu Dhabi P.O. Box 92510, United Arab Emirates

**Keywords:** multicentric breast cancer, multifocal breast cancer, breast-conserving surgery, oncoplastic surgery, magnetic resonance imaging, narrative review

## Abstract

Multicentric breast cancer—two or more biopsy-proven tumor foci in anatomically distinct regions of the same breast—has historically been treated as a near-automatic indication for mastectomy, reflecting concerns about occult residual disease, achievability of negative margins at every focus, and unacceptable cosmetic outcome. This narrative review synthesizes the evidence underlying that historical position and the more recent data that have begun to qualify it. The single-arm ACOSOG Z11102 (Alliance) trial reported a 5-year local recurrence rate of 3.1% after breast-conserving therapy for two to three ipsilateral foci and recent retrospective cohorts report comparable to local control. These findings sit alongside a persistent and unresolved tension around preoperative MRI, which improves detection of additional foci but has not demonstrated improvement in local control or survival outcomes in randomized trials, despite increasing mastectomy conversion. We review the definitions and biology of multifocal and multicentric disease, the maturing role of oncoplastic technique, margin and radiotherapy planning considerations specific to multicentric resection, the evolving use of neoadjuvant systemic therapy, hereditary cancer considerations, and the current guideline landscape (ASBrS, NCCN, ESMO, AGO). We propose a practical, multidisciplinary framework for patient selection and identify the principal gaps—absence of randomized comparison with mastectomy, unresolved necessity of routine MRI, and limited data on breast conservation after neoadjuvant therapy specifically in multicentric disease—that should guide both clinical decision-making and future research.

## Simple Summary

When breast cancer appears as two or more separate tumors in the same breast—called multicentric breast cancer—doctors have traditionally recommended removing the whole breast (mastectomy), out of concern that breast-conserving surgery could leave cancer behind or give a poor cosmetic result. This review looks at newer evidence, including a major clinical trial, showing that breast-conserving surgery can achieve low rates of cancer coming back in carefully selected patients with two or three tumor sites, when combined with modern reconstructive (oncoplastic) techniques and radiotherapy. We also discuss the role of MRI scans before surgery, which find more tumor sites but have not been shown to improve survival and can lead to more mastectomies than necessary. We summarize current international guidelines and propose a practical framework to help patients and their doctors decide, together, whether breast conservation is a safe option—while highlighting the key questions that still need to be answered by future research.

## 1. Introduction

Multicentric breast cancer has for decades been managed as a near-automatic indication for mastectomy. This position was never based on local recurrence risk in isolation; it reflected three intersecting concerns that were each real limitations of practice at the time—an inability to reliably exclude occult disease outside the resected specimen using the imaging available, a correspondingly higher risk of leaving a positive margin at one or more foci, and an inability to remove multiple tumour sites from one breast without producing a cosmetically unacceptable result [1,2]. Each of these three limitations has been substantially, though not completely, addressed by developments of the past two decades: cross-sectional imaging with MRI, oncoplastic reconstructive technique, and biologically tailored systemic therapy. This review examines the evidence behind the historical position, the evidence that has begun to qualify it, and the areas where the two remain in genuine tension. The current question is no longer whether multicentric disease can ever be treated with breast conservation, but rather which patients can safely undergo conservation without compromising oncological outcomes.

## 2. Methods

This narrative review was conducted in accordance with the SANRA (Scale for the Assessment of Narrative Review Articles) recommendations, a checklist-based framework for appraising and reporting non-systematic reviews across dimensions such as justification of the review’s importance, statement of aims, description of the literature search, and referencing quality. PubMed/MEDLINE, Embase, and Google Scholar were searched for English-language publications from January 2000 to February 2026 using combinations of the terms “multicentric breast cancer,” “multifocal breast cancer,” “multiple ipsilateral breast cancer,” “breast-conserving surgery,” “oncoplastic surgery,” “extreme oncoplasty,” “breast MRI,” “neoadjuvant systemic therapy,” and “BRCA breast conservation.” The final search was conducted on 28 February 2026. Priority was given to prospective trials, systematic reviews and meta-analyses, and current professional guideline documents; reference lists of retrieved articles were hand-searched for additional relevant studies. Titles and abstracts were screened for relevance to the surgical management of multiple ipsilateral breast cancer; full texts were retrieved where the abstract suggested original data, guideline content, or a systematic review/meta-analysis bearing directly on oncologic safety, imaging, radiotherapy planning, oncoplastic technique, neoadjuvant therapy, hereditary risk, or patient-reported outcomes in this population. Case reports, non-English-language publications, and conference abstracts without a subsequent peer-reviewed full-text publication were excluded, as were studies in which focality could not be distinguished from other reasons for mastectomy conversion. No formal risk-of-bias instrument was applied, consistent with the narrative rather than systematic design, but methodological quality—sample size, presence of a comparator group, length and completeness of follow-up, and consistency of the multifocal/multicentric definition used—was considered qualitatively in weighting how much emphasis a given study received in the synthesis. Because the great majority of the evidence reviewed here is retrospective, effect estimates for local recurrence, survival, and other oncological endpoints should be read throughout as hypothesis-supporting rather than definitive, particularly where confidence intervals are wide or where a study is subject to selection bias by treatment indication. Because this is a narrative rather than a systematic review, study selection was based on relevance to the surgical management of multicentric/multifocal disease and on methodological quality as judged by the authors, rather than on a predefined systematic search protocol or formal risk-of-bias assessment. Where evidence was sparse or conflicting—most notably regarding the necessity of routine preoperative MRI—this is stated explicitly rather than resolved by selective citation.

### Definitions, Terminology, and the Problem of Classification

Throughout this narrative review conducted in accordance with SANRA recommendations [3], multiple ipsilateral breast cancer (MIBC) is used as the umbrella term for two or more tumour foci within the same breast, encompassing both multifocal (MF) and multicentric (MC) presentations; the two subtypes are distinguished below, but no consensus definition separates them precisely, and the labels should not be read as implying a specific anatomical or prognostic meaning. Multifocal (MF) and multicentric (MC) breast cancer are frequently discussed together as “multiple ipsilateral breast cancer” (MIBC), a term increasingly preferred in the trial literature because the distinction between the two entities is not as settled as textbook definitions imply. In most contemporary usage, multifocal disease refers to two or more tumour foci within the same breast quadrant, and multicentric disease to foci in different quadrants; some groups instead define the two by an inter-tumour distance threshold (commonly cited thresholds cluster around 4–5 cm, though this figure is not standardised and different studies have used values from 1 cm to 5 cm). Neither the quadrant-based nor the distance-based definition has achieved universal consensus, and both are acknowledged in the literature to be anatomically somewhat arbitrary, since breast tissue has no true internal quadrant boundaries and lymphatic drainage does not respect them. In other words, breast quadrants are a surgical and radiological convenience for describing tumour location, not a biological or anatomical unit—a distinction that matters clinically, since resection planning, margin assessment, and mastectomy-conversion decisions are often framed in quadrant-based language despite that lack of biological grounding. This is not a semantic issue only: it is the principal reason reported incidence of MF/MC disease spans such a wide range in the literature—from roughly 10% to over 60% of breast cancers [4]—depending on whether ascertainment is by clinical examination, mammography, MRI, or whole-organ pathological sectioning, and on whether in situ lesions are counted as qualifying foci. Unless stated otherwise, the studies discussed in this review required at least one biopsy-proven invasive focus for eligibility; where in situ-only lesions were counted toward MIBC status, this is noted explicitly, since doing so measurably inflates reported incidence and is not applied consistently across the literature.

A related definitional question is whether pure DCIS foci—multiple sites of in situ disease without an invasive component—should be treated as equivalent to invasive multifocality when estimating local recurrence risk. The evidence here is more direct than for invasive disease: in a retrospective cohort of 615 women treated with breast-conserving surgery for DCIS, multifocality independently predicted local recurrence among women who did not receive adjuvant radiotherapy (10-year local recurrence-free survival 59% versus 80% for unifocal disease), but this detrimental effect was abolished once whole-breast radiotherapy was added, with no significant difference between multifocal and unifocal DCIS (80% versus 87%) and no association between multifocality and subsequent invasive recurrence [5]. This supports breast conservation as a reasonable option for multifocal pure DCIS provided radiotherapy is delivered but should not be extrapolated uncritically to invasive multifocal/multicentric disease, where systemic as well as local recurrence risk is relevant. A separate and more common clinical scenario—an invasive focus accompanied by a substantial in situ component, sometimes described as an extensive intraductal component—is associated with higher rates of positive margins and local recurrence after breast conservation [6], and is addressed further in the discussion of margin standards below.

Multifocal disease has traditionally been assumed to represent intraductal or intramammary spread from a single tumour, and multicentric disease to represent independent, biologically distinct primaries. Molecular data over the past decade complicates this binary. Targeted and whole-exome sequencing studies of paired foci from the same breast have found that a meaningful proportion of multifocal cases show strong clonal relatedness across foci, consistent with intramammary spread, while others show discordant driver mutations and copy-number profiles consistent with independent origin [7]—and this heterogeneity is not fully predicted by the anatomical (same-quadrant vs. different-quadrant) classification. A targeted-sequencing series of 21 patients with multiple ipsilateral lesions found discordant intrinsic subtype in 10% of cases and copy-number differences in a third, despite closely matched histology between foci [8]—indicating that biomarker and genomic heterogeneity between foci can occur even when the lesions look identical under the microscope. This growing biological literature is one reason quadrant-based nomenclature is increasingly regarded as a surgical planning convenience rather than a biological classification, and why some authors now argue for describing this population primarily as MIBC rather than forcing every case into an MF/MC dichotomy.

Whole-organ pathological sectioning studies, dating from the 1980s and 1990s, found occult multicentric foci in a large proportion of mastectomy specimens even when preoperative assessment had suggested unifocal disease [9,10]—one frequently cited whole-organ series found multicentric foci beyond the index quadrant in nearly 80% of breasts that harboured any additional foci at all [9]. The clinically important counterpoint, drawn from long-term breast-conservation follow-up series, is that the great majority of true local recurrences occur at or near the index tumour bed rather than in these unsampled additional foci, suggesting that pathological multicentricity [11] and clinically meaningful local recurrence risk are not the same thing. This distinction is central to understanding why breast conservation can be oncologically reasonable in selected multicentric disease despite the historical concern about undetected additional foci.

## 3. Results

### 3.1. The Historical Case for Mastectomy

Cohort studies auditing reasons for exclusion from breast conservation have consistently identified multifocal or multicentric disease as the single most common disqualifying factor, ahead of diffusely abnormal mammographic findings and unfavorable tumor-to-breast-volume ratio. Early pooled analyses supported treating multicentricity with caution: a 2014 systematic review and meta-analysis found higher local recurrence after breast-conserving surgery in multifocal/multicentric disease compared with unifocal cancer [12], though this signal was driven predominantly by cohorts treated before 2000, before the routine use of adjuvant systemic therapy, modern radiotherapy planning, and oncoplastic reconstruction were established practice. This body of evidence is the origin of language still found in current guidance—the American Society of Breast Surgeons’ resource guide, for example, continues to list multicentric disease not amenable to oncoplastic surgery among the absolute contraindications to breast conservation [13]—even as the same documents increasingly qualify that position, as discussed later in this article.

A less commonly articulated but clinically important corollary of this history is that mastectomy is not itself risk-free. Beyond the immediate surgical burden, mastectomy carries its own consequences relative to breast conservation—loss of native breast sensation, the additional morbidity and potential complications of reconstruction, delays to adjuvant therapy where reconstruction is complex, and measurable psychological effects on body image and quality of life. The clinical argument here is not that mastectomy should be avoided in multicentric disease; it is that mastectomy should not be offered on the basis of an indication that reflects outdated assumptions about achievable margins, imaging accuracy, or cosmetic feasibility rather than a contemporary, individualized assessment. Where oncoplastic technique, current imaging, and modern systemic therapy have closed the gap that originally justified reflexive mastectomy, continued routine use of mastectomy in all such cases may represent persistence of historical practice patterns rather than evidence-based individualization.

### 3.2. The Evidence Base for Breast Conservation in Multicentric Disease

No single study settles the question of oncologic safety, and the evidence is best read as several independent lines that converge on a broadly consistent, though not unanimous, conclusion.

**Prospective trial evidence.** ACOSOG Z11102 (Alliance) remains the only prospective trial to address this question directly. It enrolled 270 women aged 40 years and older with two to three biopsy-proven, cN0–N1 ipsilateral foci, each separated by at least 2 cm of normal tissue and confined to two quadrants, treated with lumpectomy to negative margins followed by whole-breast radiotherapy with a boost to every lumpectomy bed; neoadjuvant therapy was not permitted, and preoperative MRI, initially mandatory, was later made optional. Among 204 evaluable patients, the 5-year cumulative incidence of local recurrence was 3.1% (95% CI 1.3–6.4%), below the prespecified acceptability threshold of 8%. Adherence to endocrine therapy in hormone-receptor-positive patients was associated with lower local recurrence; age, number of preoperative biopsy sites, and HER2 status were not independent predictors [2]. As a single-arm design without a mastectomy comparator, the trial cannot quantify relative risk against mastectomy directly, and its statistical framing drew published commentary and a formal reply from the investigators [14].

**Imaging-focused prospective data.** The MIPA (Multicenter International Prospective Analysis) study, a large prospective cohort examining how screening and diagnostic breast MRI affect surgical treatment, provides complementary evidence [15] on how imaging strategy—rather than tumor biology—shapes the surgical pathway in this population; its findings are discussed together with other MRI trials later in this review, since they speak primarily to the imaging question rather than to oncologic safety of conservation itself.

**Oncoplastic comparative data.** A retrospective matched cohort study compared 100 patients with multicentric, or multifocal tumors treated with oncoplastic breast-conserving surgery against 100 matched patients treated with mastectomy. Overall and disease-free survival were similar between groups; local events were somewhat more frequent after oncoplastic surgery and regional events somewhat more frequent after mastectomy, but neither difference reached statistical significance, and distant recurrence was similar [16]. The authors characterized this as the best available evidence to date supporting oncoplastic breast conservation in this population, while explicitly calling for randomized confirmation [16].

Retrospective single-center cohorts. Two recently reported series add real-world texture using different selection strategies. A retrospective Argentinian cohort found breast conservation feasible and oncologically comparable in stage I–II multifocal/multicentric disease (n = 91, 7.7% of the overall cohort) versus unifocal disease (n = 1097): 5-year locoregional recurrence was 3.3% versus 5.9%, with similar distant recurrence (3.3% vs. 4.8%). This cohort achieved these outcomes without routine preoperative MRI and excluded patients who received neoadjuvant chemotherapy [17]. A Turkish single-center series identified 246 of 2155 patients with multifocal/multicentric disease over a recent three-year period, of whom 68 underwent breast conservation (27.6% of the MF/MC group); among all 1253 patients undergoing breast-conserving surgery, these 68 represented 5.4%. Negative margins were achieved in 90% at first surgery; five patients (7.4%) required re-excision, and two (2.9%) ultimately required completion mastectomy. The overall complication rate was 19%, and 90% of 46 surveyed patients rated their cosmetic outcome as excellent or good. Unlike the Argentinian cohort, this series used routine preoperative MRI and structured multidisciplinary evaluation [18].

**Systematic reviews.** A 2022 systematic review and meta-analysis concluded that multicentric/multifocal breast cancer does not universally carry worse survival than unifocal disease [19], although some included analyses suggested a small increase in mortality odds that the authors attributed at least partly to confounding by stage and molecular subtype. Methodological heterogeneity across the underlying studies—inconsistent definitions of multicentricity, variable radiotherapy boost practice, and differing margin standards—was identified as a limiting factor across this literature, a caveat echoed in a separate 2025 narrative synthesis of more than fifty studies on this topic [20].

**Comparative meta-analysis.** The most direct synthesis to date is a 2026 systematic review and meta-analysis that pooled study-level risk ratios for BCS versus mastectomy specifically in MIBC, rather than comparing multifocal/multicentric against unifocal disease [21]. It included 17 comparative studies—16 retrospective and one nominally randomized series that was analyzed as non-randomized because of incomplete reporting of allocation and intention-to-treat methods—comprising 29,711 patients (9587 BCS, 20,124 mastectomy) drawn mainly from European and North American cohorts published between 1999 and 2025, with a median follow-up of 62 months and a median Newcastle–Ottawa quality score of 8 out of 9. Pooled analysis found no significant difference between BCS and mastectomy in local recurrence (RR 1.06, 95% CI 0.74–1.51), locoregional recurrence (RR 1.02, 95% CI 0.68–1.52), distant recurrence (RR 0.78, 95% CI 0.54–1.12), or disease-free survival (RR 0.82, 95% CI 0.60–1.13). Overall survival significantly favored BCS in the primary analysis (RR 0.60, 95% CI 0.43–0.82), but this advantage was not maintained in a sensitivity analysis restricted to upfront-surgery cohorts (RR 0.77, 95% CI 0.31–1.91), suggesting the survival signal is more plausibly explained by selection and treatment heterogeneity—particularly inconsistent inclusion of neoadjuvant systemic therapy across studies—than by a genuine oncological advantage of breast conservation. The authors themselves highlight important limitations: the evidence base remains almost entirely retrospective and heterogeneous in its definitions of multifocality and multicentricity, margin standards, and radiotherapy technique; the single largest contributing cohort supplied overall-survival data only and was excluded from the recurrence and disease-free-survival analyses—by our reading of the study-level numbers in the source publication, this one cohort accounted for roughly 90% of the patients contributing to the overall-survival analysis specifically [22], so the pooled overall-survival estimate is driven overwhelmingly by a single registry dataset rather than corroborated independently across the 17 included studies; and subgroup analysis by MF versus MC status, or by higher-risk biology (HER2-positive and triple-negative disease, tumors larger than 5 cm, or three or more foci), was not possible. These limitations temper, without negating, the reassuring headline findings, and a leave-one-out or influence analysis excluding this dominant cohort would help clarify how much of the overall-survival signal survives its removal. Readers should not interpret the overall-survival finding as evidence that BCS is oncologically superior to mastectomy in MIBC; the more defensible reading is that BCS is not inferior to mastectomy for oncological safety in appropriately selected patients, not that it is the preferable option.

A further source of heterogeneity between the neoadjuvant and upfront-surgery analyses deserves consideration: patients receiving neoadjuvant systemic therapy almost universally undergo preoperative breast MRI for staging, response assessment, and surgical planning, whereas MRI utilization among patients treated with upfront surgery is considerably more variable. In the Alliance ACOSOG Z11102 trial [2], an exploratory subgroup analysis found a substantially lower 5-year local recurrence rate among patients who underwent preoperative MRI compared with those who did not (1.7% vs. 22.6%) [2]. Although this analysis was neither randomized nor prespecified and should therefore be regarded as hypothesis-generating, it raises the possibility that differences in MRI utilization—and consequently in patient selection for breast-conserving surgery—may have contributed to the heterogeneity observed between analyses that include neoadjuvant cohorts and those restricted to upfront surgery. This same imaging-driven staging heterogeneity plausibly bears on the overall-survival finding specifically, not only on local recurrence: because patients receiving neoadjuvant therapy are almost uniformly staged with MRI, the neoadjuvant-inclusive analyses may reflect a more consistently and accurately characterized multifocal/multicentric population than the upfront-surgery sensitivity analysis, where variable MRI use raises the possibility that some patients were imprecisely classified; were preoperative MRI applied uniformly across the upfront-surgery cohorts, the overall-survival advantage associated with BCS might plausibly emerge as a more consistent finding rather than the attenuated signal currently observed—consistent with separate evidence from the neoadjuvant setting, where breast conservation with radiotherapy has been associated with improved overall survival compared with mastectomy in a cohort staged with the more uniform imaging that neoadjuvant pathways entail [23]. Future comparative studies should consider preoperative imaging strategy, alongside systemic therapy and radiotherapy approach and dose/boost recommendations, as potential confounders when evaluating the oncological outcomes of BCS in multiple ipsilateral breast cancer. This aligns with current guideline recommendations that MRI is most valuable when multicentricity is already suspected, and breast conservation is being actively considered, rather than used universally for all patients.

Read together, these independent sources—one prospective single-arm trial, one prospective imaging cohort, one matched retrospective comparison against mastectomy, two real-world single-center series using different imaging strategies, pooled systematic-review data, and the largest comparative meta-analysis to date—converge on the conclusion that carefully selected patients can achieve local recurrence rates in the low single digits at 5 years and oncological outcomes broadly comparable to mastectomy, but none individually, nor their combination, replaces the randomized comparison that is still absent from this literature. It is also worth emphasizing that virtually all of this evidence—including Z11102 and the pooled meta-analysis above—concerns disease with two or three ipsilateral foci; data supporting breast conservation for four or more separate foci are essentially absent, and this should be treated as a de facto boundary for patient selection outside highly individualized, oncoplastically favorable cases.

The apparent equivalence between BCS and mastectomy across this evidence base should not be interpreted as evidence that the two procedures are interchangeable for all patients. Patients selected for BCS are not simply healthier; they are selected on the basis of favorable surgical anatomy—breast size, tumor-to-breast-volume ratio, lesion distribution and inter-focal distance, lesion accessibility, MRI findings, surgeon expertise, and patient preference—variables that influence both whether BCS is performed and the oncological outcomes subsequently observed. Current evidence therefore reflects outcomes among patients already judged suitable for conservation, creating an unavoidable confounding by indication that no amount of retrospective adjustment can fully resolve; this is a structural limitation of the evidence base itself, not merely of any one study within it. The key characteristics of the principal studies underlying the evidence base are summarized in Table 1.

### 3.3. Preoperative Imaging: A More Selective Role for MRI than Sometimes Assumed

Breast MRI has the highest sensitivity of any modality for detecting additional occult foci, and this is both its principal value and the source of its principal controversy in this context. Meta-analyses have shown that MRI identifies additional malignant lesions, and correspondingly changes the surgical plan, more often than mammography and ultrasound alone [24]; the MIPA cohort demonstrates this effect prospectively at scale [15]. However, a meaningful proportion of MRI-detected additional findings are false positive on subsequent biopsy or histology—in the COMICE trial, roughly a third of MRI-prompted mastectomies were later found to have no malignancy in the additional tissue removed [25]. Randomized evidence is more circumspect still: The BREAST-MRI trial, which randomized 524 women eligible for breast conservation to preoperative MRI or standard imaging, found that MRI increased the initial mastectomy rate from 0.4% to 8.3%, without measurable improvement in 5-year local relapse-free survival, overall survival, or reoperation rates [26].

The exploratory subgroup finding from Z11102 is often cited as evidence that MRI should be routine in this population: patients not imaged with MRI had a 5-year local recurrence of 22.6% versus 1.7% in those who were [2], but this comparison was neither randomized nor prespecified, involved a small number of unimaged patients, and cannot exclude confounding by indication (patients selected for surgery without MRI may have differed in other clinically relevant ways). This finding is best regarded as hypothesis-generating rather than definitive proof that MRI is mandatory in this population, particularly given that adequately powered randomized trials of preoperative MRI have shown no corresponding survival benefit. Current guideline language, and the balance of evidence reviewed here, supports a more selective position than “MRI should be done” or “MRI should be routine for everyone”: MRI is most useful, and most likely to change management appropriately, in patients where multicentricity is already suspected or confirmed and breast conservation is being actively considered—that is, precisely the population under discussion in this review—rather than as a reflexive addition to every preoperative workup. Critically, any additional lesion identified on MRI should be confirmed histologically before it is allowed to alter the surgical plan; imaging findings alone, in the absence of biopsy confirmation, should not convert a patient from a breast-conservation to a mastectomy candidate.

### 3.4. Margin Assessment in Multicentric Resection

The margin standard for invasive breast cancer treated with whole-breast irradiation—“no ink on tumor”—was established by the 2014 SSO-ASTRO consensus guideline on the basis that wider clear margins do not further reduce local recurrence [27], and this standard applies without modification to each individual focus in a multicentric resection. In practice, this means that achieving “no ink on tumor” at every excised focus, rather than at a single dominant lesion, is the operative surgical target in multicentric disease, and multifocality/multicentricity has itself been shown in retrospective series to be independently associated with higher rates of positive margins and higher conversion to mastectomy at first surgery [28]—a technical, rather than purely oncologic, argument for the additional value of oncoplastic planning and, in some cases, of intraoperative margin assessment. An important distinction is that the 2 mm margin threshold from the 2016 SSO-ASTRO-ASCO consensus guideline applies specifically to pure DCIS treated with whole-breast irradiation, in the absence of an invasive component. Where DCIS accompanies invasive disease at the same focus, the 2014 SSO-ASTRO “no ink on tumor” standard governs both the invasive and the DCIS margins at that focus, and the two thresholds should not be applied selectively within a single specimen. Re-excision of a close, uninvolved DCIS margin in this mixed setting is not automatic and should be individualized according to the extent of residual DCIS, its proximity to the inked margin, and the overall pathological picture, rather than driven by a fixed millimeter threshold derived from a pure-DCIS population.

### 3.5. Oncoplastic Techniques: Making Multicentric Conservation Feasible

The principal practical enabler of breast conservation in multicentric disease has been the maturation of oncoplastic technique. As defined by the ASBrS, oncoplastic breast surgery combines an oncologic partial mastectomy with immediate repair of the resulting defect using volume-displacement or volume-replacement reconstructive technique [29]. Volume-displacement approaches (local tissue rearrangement, therapeutic mammaplasty, Wise-pattern reduction with contralateral symmetrization) and volume-replacement approaches (pedicled or perforator flaps, including intercostal artery perforator flaps) allow resection of two or more separate tumor beds—in some series removing 20–50% or more of breast volume in aggregate—while preserving contour, nipple-areolar position, and symmetry with the contralateral breast. “Extreme oncoplastic” series applied specifically to multifocal/multicentric and locally advanced disease, in patients with adequate breast volume, report favorable BREAST-Q outcomes alongside acceptable margin rates, extending eligibility to patients who would previously have been offered only mastectomy [30]. This is consistent with AGO guidance, which states that oncoplastic surgery is oncologically safe and carries complication rates comparable to standard breast-conserving surgery, and on this basis recommends that oncoplastic technique can replace mastectomy across several indications, including multicentric or multifocal tumors [31,32]. Anatomically demanding sites—particularly the upper inner and central/retro areolar quadrants, where local tissue mobility is limited—remain the most technically challenging scenarios for multicentric conservation and are the subject of an active case-series and technique-development literature, and cases involving these sites are often best referred to specialist oncoplastic centers rather than managed as routine multicentric resections, reflecting that oncoplastic feasibility, rather than oncologic principle alone, is often the practical rate-limiting factor in offering breast conservation for multicentric disease. As above, the matched-cohort data from De Lorenzi et al. provide the strongest currently available comparative evidence that this approach does not compromise survival relative to mastectomy [16], while the authors themselves note that a double radiotherapy boost after double lumpectomy cavities, and other technical questions specific to multicentric oncoplasty, remain without prospective, randomized evidence. Delivering this approach at scale also has resource implications worth acknowledging for guideline implementation: multicentric oncoplastic resection, double-boost radiotherapy planning, and multidisciplinary case discussion require specialist-center capacity and training that is not uniformly available, which may itself be a rate-limiting factor independent of oncologic or technical feasibility.

Selecting an oncoplastic approach for multisite resection begins with quantifying two variables: the volume to be excised relative to total breast volume, and the anatomical difficulty of each tumor bed. The widely used Clough classification, subsequently adopted by the ASBrS consensus definition, stratifies oncoplastic volume-displacement surgery into two tiers on this basis: level I procedures (glandular mobilization and local rearrangement, without a formal skin-reduction pattern) accommodate resection of up to approximately 20% of breast volume, while level II procedures (therapeutic mammaplasty or Wise-pattern reduction with contralateral symmetrisation) extend to resections of 20–50% of breast volume [33,34]. In multisite disease this threshold is applied cumulatively across all planned excision sites rather than to each focus individually, since it is the aggregate parenchymal deficit—not any single tumor—that determines whether local tissue rearrangement alone can achieve an acceptable contour. When the combined resection volume of two or more foci is estimated to exceed approximately 50% of breast volume, or when the patient has a small-to-moderate breast with limited glandular reserve, volume-replacement technique—a pedicled or perforator-based flap such as the lateral intercostal artery perforator (LICAP), thoracodorsal artery perforator (TDAP), or latissimus dorsi mini-flap—is generally required in preference to further volume displacement, to avoid contour deformity [35].

Beyond aggregate volume, individual quadrant anatomy independently constrains what a given technique can achieve, and this is particularly consequential in multicentric disease, where two or more quadrants are frequently involved simultaneously. The upper inner and central/retro-areolar quadrants have the least mobile, most adherent parenchyma and the smallest margin for local rearrangement before nipple–areola complex (NAC) malposition or contour step-off becomes visible; series specific to this location report that standard inferior-pedicle or batwing mastopexy technique is often inadequate once resection at this site alone approaches 15–20% of breast volume, favoring an extended pedicle, matrix-rotation flap, or pedicled pectoralis major myofascial flap instead. The lower pole and lateral quadrants tolerate proportionally larger resections—up to, and sometimes beyond, the level II threshold—using superior or superomedial pedicle mammaplasty, reflecting greater tissue mobility and a more forgiving aesthetic unit. Table 2 summarizes technique selection by quadrant and estimated resection volume, synthesizing the quadrant-per-quadrant atlas approach with the level I/II volume framework, for use in preoperative planning of multisite resection [33,34,35]. Two practical corollaries follow for multisite disease specifically: first, when two foci lie in adjacent quadrants sharing a single subdermal and glandular blood supply, they can often be excised through a single, shared oncoplastic pedicle rather than two independent procedures, reducing cumulative devascularization risk; second, when foci lie in non-adjacent or bilaterally opposed quadrants (for example, upper-outer and lower-inner), independent pedicles are required for each defect, and the aggregate—not the larger single-focus—resection volume should guide the level I/II decision described above.

### 3.6. Radiotherapy Planning Considerations

Z11102’s protocol—whole-breast irradiation with a boost delivered to every lumpectomy bed—is the only prospectively tested radiotherapy approach in this population and is the de facto standard reflected in most subsequent series [2]. Several technical questions remain unresolved and are addressed inconsistently across the literature: whether two separate cavity boosts are needed when lumpectomy sites are close together versus widely separated within the breast, how cavities should be localized and delineated when more than one resection has occurred (clip placement at the time of surgery is widely used for this purpose but is not standardized across the multicentric-disease literature specifically), and whether cumulative boost dose to overlapping treatment volumes carries additional toxicity. In the absence of dedicated randomized data, radiotherapy planning for multicentric resection is generally extrapolated from single-site boost practice, and this extrapolation—while clinically reasonable—is acknowledged in the oncoplastic and radiation oncology literature as a genuine, not merely theoretical, gap. No dedicated ASTRO or SSO-ASTRO consensus statement governs multi-cavity boost specifically, and current practice for closely spaced or overlapping cavities is extrapolated from general conformal planning principles rather than disease-specific guidance: where two lumpectomy beds lie close together, a single merged boost volume encompassing both cavities is commonly used, whereas widely separated cavities are more often treated with two discrete boosts, increasingly delivered by intensity-modulated or volumetric-modulated arc therapy to improve conformity and limit dose to the intervening normal breast tissue [20]. Whether cumulative dose to breast parenchyma between two boosted cavities translates into measurably higher rates of fibrosis or other late toxicity has not been formally tested in this population and remains guided by extrapolation and institutional experience rather than trial data.

### 3.7. Neoadjuvant Systemic Therapy and Response-Guided Surgery

Neoadjuvant systemic therapy (NST) has reshaped surgical planning in breast cancer generally, and multicentric disease is no exception. For biologically aggressive subtypes—triple-negative and HER2-positive disease in particular—and for patients with an unfavorable tumor-to-breast-volume ratio or multifocal/multicentric disease, NST offers the opportunity to downstage individual foci and convert patients who would otherwise require mastectomy into breast-conservation candidates; professional guidance now explicitly encourages consideration of NST for this purpose [29]. Practical surgical planning around NST in this population depends on response-guided principles rather than a fixed protocol: imaging (mammography, ultrasound, and MRI) is repeated after treatment to reassess the extent and distribution of residual disease at each focus; radio-opaque or other localization clips are placed in each individual tumor bed before treatment begins, since foci that respond well—including those achieving a complete radiological response—can otherwise become impossible to localize for targeted excision; and final resection is planned around the post-treatment extent of residual disease at each site rather than the pretreatment extent of disease, provided each original clip-marked site remains excisable to a negative margin.

Confirming that all disease has been removed depends on more than imaging and clip placement alone: intraoperative specimen radiography of each excised focus, correlation between the radiological and pathological extent of disease at each site, and explicit radiology-pathology discussion at multidisciplinary meeting are practical safeguards against under-excision in multicentric resection, and take on particular importance after neoadjuvant therapy, when the post-treatment radiological appearance may understate residual viable tumor at an individual focus.

The evidence base specific to breast conservation after NST in multiple ipsilateral breast cancer is smaller than for upfront surgery—Z11102 itself excluded patients who received neoadjuvant therapy—though a large cohort from the GeparTrio, GeparQuattro, and GeparQuinto neoadjuvant chemotherapy trials (n = 6134) has examined the impact of focality on surgery type and survival outcomes after NST [36]. In that cohort, 820 patients (13.4%) had multifocal and 581 (9.5%) had multicentric disease, with pathological complete response rates of 16.5% and 14.4% respectively; among patients who did not achieve a pathological complete response, those with multicentric disease had significantly worse local recurrence-free survival than patients with unifocal disease, whereas no such difference was seen once a pathological complete response or breast conservation with negative margins was achieved—indicating that response and margin status, rather than focality alone, are the operative predictors after NST. The published analysis did not report this outcome stratified by molecular subtype, which remains a specific gap for future analyses of this and comparable neoadjuvant cohorts [36], and more recent single-institution series addressing breast conservation specifically after NST in this population are only beginning to report outcomes. Guideline bodies remain correspondingly cautious: AGO guidance explicitly notes that multicentricity may still justify mastectomy even when a good response to neoadjuvant therapy has been achieved at each focus [31], reflecting persistent uncertainty about whether a favorable radiological or pathological response reliably predicts durable local control after conservation in this specific population. This caution is not unique to AGO—it reflects a broader evidence gap shared across guideline bodies, since long-term local-control data specific to breast conservation after a good neoadjuvant response in multicentric disease remain sparse regardless of which group is asked.

### 3.8. Hereditary Breast Cancer: A Distinct Consideration

Multicentricity does not exist in isolation from hereditary cancer risk, and the two considerations should not be conflated in surgical planning. Pathogenic variants in BRCA1, BRCA2, PALB2, and TP53 are associated with an increased likelihood of multicentric and bilateral disease, and germline testing for this panel is indicated in this population according to standard clinical and family-history criteria regardless of whether the presenting tumor is unifocal or multicentric—though, as discussed below, a TP53 result is managed very differently from the other three genes. Among BRCA1/BRCA2 carriers who undergo breast-conserving therapy, cohort data show a higher rate of ipsilateral breast events compared with non-carriers—a landmark comparative series reported a 15-year cumulative ipsilateral risk of 23.5% after breast conservation versus 5.5% after mastectomy [37]—without a corresponding difference in overall survival, since most subsequent ipsilateral events in carriers represent new primary cancers rather than true recurrences of the treated tumor. Framed this way, the relevant clinical conversation for a BRCA carrier with multicentric disease is not whether breast conservation is oncologically unsafe—the survival data do not support that—but how much residual, largely new-primary risk the patient is willing to accept relative to the more radical risk reduction offered by mastectomy. This means the decision between breast conservation and mastectomy in a BRCA carrier with multicentric disease is properly framed as a risk-reduction and shared decision-making discussion—encompassing contralateral risk-reducing mastectomy, surveillance intensity, and the patient’s own risk tolerance—rather than as a strictly oncologic determination based on the index tumor’s focality alone.

TP53 warrants separate discussion because it is managed differently from BRCA1, BRCA2, and PALB2 at the point of locoregional treatment selection, not only at the level of screening. TP53 pathogenic variants (Li–Fraumeni syndrome) confer both a very high lifetime breast cancer risk and increased cellular radiosensitivity, with a recognized risk of radiation-induced second primary malignancies—including within a previously irradiated field. Current guidance therefore favors mastectomy over breast-conserving surgery specifically to avoid adjuvant radiotherapy in confirmed TP53 carriers, in contrast to BRCA1/BRCA2/PALB2 carriers, for whom breast conservation remains an oncologically reasonable option and radiotherapy is not itself contraindicated [38]. This distinction has a direct, practical consequence for multicentric disease: a patient with multiple foci who might otherwise be a borderline oncoplastic candidate should not be carried through the margin-and-cosmesis feasibility assessment described below if a TP53 pathogenic variant is identified or strongly suspected, since the basis for excluding breast conservation in that scenario is the radiotherapy contraindication itself, independent of whether all foci could technically be excised to a negative margin. Confirmatory germline testing should therefore be expedited, and ideally completed before the final surgical decision, whenever clinical or family-history criteria raise specific suspicion of Li–Fraumeni syndrome rather than isolated BRCA1/BRCA2/PALB2-related risk.

### 3.9. The Current Guideline Landscape

No major guideline body has abandoned caution around multicentric disease, but the direction of travel across guidelines is consistent: from a categorical contraindication toward a conditional, multidisciplinary pathway. The current positions of the major guideline bodies are summarized in Table 3.

The practical convergence across these frameworks is that imaging adequacy, achievability of negative margins at every focus, and cosmetic feasibility function as the operative gatekeeping criteria, applied through multidisciplinary discussion rather than a fixed anatomical rule.

A further point deserves emphasis: guideline caution and clinical equivalence are not the same thing. Guideline bodies appropriately retain caution around multicentric disease because prospective comparative data against mastectomy remain absent, and this caution is a defensible response to genuine uncertainty. However, that caution should not be read as evidence that the historical, categorical contraindication and a contemporary, evidence-based selective approach carry equal clinical validity—the retrospective and pooled evidence reviewed here has moved materially beyond the assumptions that originally justified reflexive mastectomy, even though no guideline has yet been able to formalize that shift into a prescriptive rule. Guideline conservatism, in other words, reflects the absence of a particular kind of evidence (randomized comparison) rather than the presence of evidence against conservation.

### 3.10. Patient-Reported Outcomes and the Cosmetic Dimension

Cosmesis is intrinsic to the rationale for offering conservation at all in this population—if multicentric excision routinely produced unacceptable deformity, the argument for avoiding mastectomy would be considerably weaker regardless of local recurrence data. The Z11102 cosmesis sub-study, using a four-point cosmesis scale and the BREAST-Q, found patient- and surgeon-reported cosmetic outcomes after multicentric lumpectomy and whole-breast radiation broadly comparable to single-site breast conservation [39], and extreme-oncoplastic series reports similarly favorable BREAST-Q results [30]. However, formal comparative patient-reported outcome data specific to multicentric resection remain limited relative to the volume of oncologic outcome data; most published series are single-center, lack a contemporaneous mastectomy-with-reconstruction comparator, and report outcomes at relatively short follow-up, which limits how confidently patients can currently be counselled on relative cosmetic outcomes between the two approaches.

Data specific to long-term sequelae beyond cosmesis—chronic pain, sensory disturbance, and psychosocial or sexual well-being—are sparser still. The one study to compare quality of life directly between unifocal and multifocal/multicentric disease after breast-conserving surgery and radiotherapy found that the presence of multiple tumor sites did not itself worsen late cosmetic outcome or quality of life; rather, quality of life was driven mainly by the extent of the radiotherapy boost volume and the addition of regional nodal fields, irrespective of focality [40]. This suggests that any excess burden of chronic pain or sensory change after multicentric resection may be attributable more to treated volume than to multifocality or multicentricity per se, but this remains an inference from a single retrospective series rather than an established finding, and dedicated, validated assessment of chronic pain, sensory change, and sexual well-being specifically in multicentric resection—ideally with a contemporaneous mastectomy comparator—is needed before patients can be counselled with confidence on these domains.

Where BREAST-Q data specific to level II (20–50% volume) oncoplastic resection have been reported—the resection extent most relevant to multisite disease—the concrete domain scores provide a useful benchmark. In a cohort of 120 women undergoing unilateral oncoplastic volume-displacement surgery (median 15% of breast volume excised, range 3–35%), the median BREAST-Q Satisfaction with Breasts score was 74/100, with lower scores independently associated with axillary clearance, neoadjuvant chemotherapy, and low breast density [41]. A separate cohort of 109 women undergoing bilateral level II volume-displacement surgery (20–50% of breast volume excised) reported an almost identical median Satisfaction with Breasts score of 74/100, with central-quadrant tumor location, triple-negative biology, and need for re-intervention each independently associated with a lower aesthetic-satisfaction index [42]—a finding of direct relevance to multisite disease, in which a central or upper-inner focus is common and in which more than one resection cavity increases the cumulative re-intervention risk that this cohort identified as a driver of lower satisfaction. Neither cohort was restricted to multicentric or multifocal disease, so these figures should be read as the closest available benchmark for level II resection volume rather than as multisite-specific data; they sharpen, rather than resolve, the evidence gap this review identifies for psychosocial and sexual well-being domains and for chronic pain specifically after multiple concurrent resection cavities.

### 3.11. Synthesis: A Practical, Shared-Decision Framework for Patient Selection

**Figure 1:**
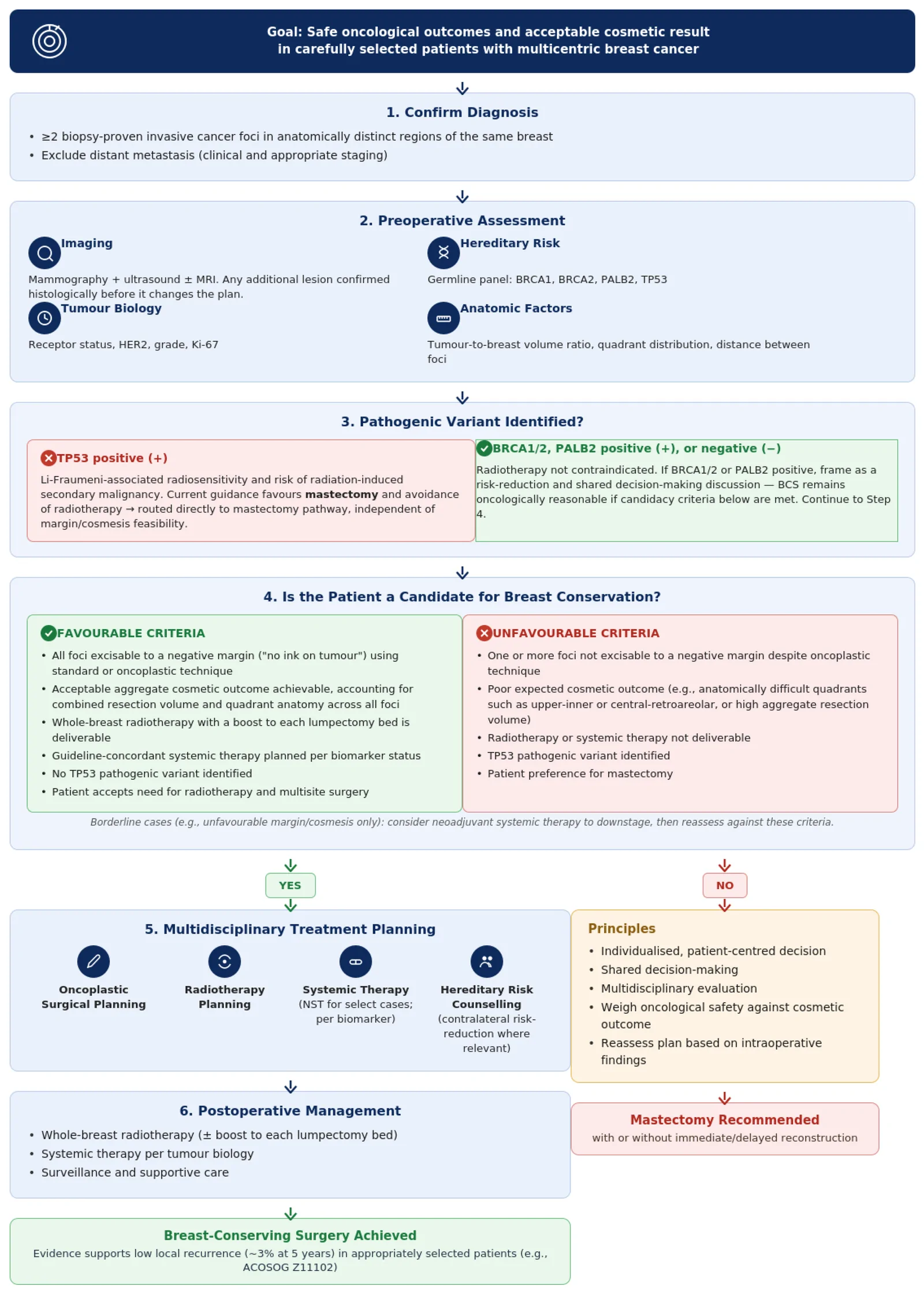
Framework for patient selection for breast-conserving surgery (BCS) in multicentric breast cancer, summarizing diagnostic confirmation, preoperative assessment, germline genetic testing (BRCA1, BRCA2, PALB2, TP53), the favorable/unfavorable candidacy criteria discussed in the text, multidisciplinary treatment planning, and postoperative management. A positive TP53 result is routed directly to the mastectomy pathway, independent of margin or cosmesis feasibility, reflecting current guidance favoring mastectomy and avoidance of radiotherapy in TP53 carriers because of radiosensitivity and radiation-induced secondary malignancy risk; a positive BRCA1, BRCA2, or PALB2 result does not preclude BCS and is instead framed as a risk-reduction and shared decision-making discussion. Candidacy is determined by the achievability of clear margins, deliverability of radiotherapy and systemic therapy, and expected cosmetic outcome—not by the number of tumor foci in isolation. BCS: breast-conserving surgery; NST: neoadjuvant systemic therapy; MDT: multidisciplinary team.

Drawing the prospective, retrospective, and guideline evidence together, five conditions recur across the literature as practical prerequisites for offering breast conservation in multicentric disease:

1. **Imaging adequacy with histologic confirmation.** Preoperative imaging—MRI included where multicentricity is suspected and conservation is being considered—has characterized the extent and distribution of disease, and any additional lesion that would change management has been confirmed histologically rather than acted upon by imaging appearance alone.
2. **Complete excision with acceptable cosmesis.** All identified foci can be excised to a negative margin (“no ink on tumor” for every invasive focus and for any DCIS coexisting with invasive disease at the same focus; 2 mm applies only to a purely in situ, DCIS-only focus) using standard or oncoplastic technique, while preserving an acceptable cosmetic result—in practice usually requiring access to oncoplastic expertise. Two practical gatekeepers deserve explicit attention: the tumor-to-breast-volume ratio, since even skilled oncoplastic technique cannot always remove multiple large-volume foci without compromising cosmesis; and anatomical site, since central/retro areolar and upper-inner-quadrant foci are technically more challenging and may warrant referral to a specialist oncoplastic center.
3. **Adjuvant radiotherapy.** Whole-breast radiotherapy with a boost to each lumpectomy bed is delivered, acknowledging that some technical aspects of multicentric boost planning remain without dedicated evidence.
4. **Guideline-concordant systemic therapy.** Endocrine, HER2-targeted, or chemotherapy is administered according to standard biomarker-driven indications, given the specific Z11102 signal linking endocrine therapy adherence to local recurrence risk [2].
5. **Shared decision-making, informed by hereditary risk where relevant.** The patient has been counselled on the comparative evidence—including its genuine limitations—and, where a pathogenic variant is identified or strongly suspected, on how that risk should inform the choice between conservation and mastectomy independent of focality. These five prerequisites are summarized in Table 4, while the overall patient-selection pathway is illustrated in Figure 1

The value of this framework is best illustrated by contrast. Two patients with multicentric disease may appropriately receive different recommendations even though both nominally meet a MIBC definition. Consider a patient with a small breast, three foci in difficult anatomical locations, and an expected cosmetic result that oncoplastic technique cannot meaningfully improve—mastectomy, with or without reconstruction, is the more appropriate recommendation. Contrast this with a patient with a larger breast, two well-separated small tumors, straightforward oncoplastic feasibility, and a good response to systemic therapy—breast conservation is oncologically reasonable and is the recommendation this review supports. Both patients carry the same anatomical label; the appropriate surgery differs. This is precisely why anatomical labels alone—multifocal, multicentric, or the number of foci in isolation—are inadequate as a basis for the conservation-versus-mastectomy decision, and why the five conditions above, applied through multidisciplinary discussion, are the more clinically meaningful unit of assessment.

When these conditions are met, the aggregate evidence reviewed here suggests local recurrence rates in the low single digits at 5 years, broadly comparable to unifocal disease treated by breast conservation—a materially different clinical picture from a categorical “multicentricity equals mastectomy” position, while still requiring more deliberate, multidisciplinary evaluation than is typical for unifocal disease.

## 4. Gaps in the Evidence and Future Directions

Several questions remain genuinely unresolved. No randomized trial has directly compared breast conservation with mastectomy in multicentric disease, and equipoise and enrolment challenges make it plausible that none ever will, meaning the field will likely continue to depend on single-arm and retrospective comparative data for the foreseeable future [21]. Although prospective randomized clinical trials remain the ideal way to confirm this, this paradigm shift is evolving rapidly, and as clinical evidence continues to accumulate alongside advances in oncoplastic surgery, it is becoming increasingly difficult to justify—or even to conduct—a randomized controlled trial in this setting. The current body of evidence provides reassuring support for the oncological safety of breast-conserving surgery in appropriately selected patients with multicentric disease. The necessity of routine preoperative MRI remains unresolved for the reasons discussed earlier in this review, and this review has deliberately not resolved that tension, since the underlying trials do not resolve it either. Technical questions in oncoplastic and radiotherapy planning—optimal boost strategy after multicentric resection, sequencing of contralateral symmetrisation, and management of anatomically difficult sites—remain addressed mainly by case series and single-center reports rather than comparative studies. Evidence on breast conservation after NST specifically in multicentric disease is the least mature of the areas reviewed here, since the pivotal prospective trial excluded these patients entirely. Finally, growing evidence of genomic heterogeneity between foci—including cases where multifocal lesions are demonstrably clonally unrelated despite matched histology [7]—raises the possibility that future classification of this disease will shift from anatomical toward biological criteria, which could refine, in either direction, which patients are genuinely appropriate for breast conservation. Such classification may ultimately determine not only surgical eligibility but also whether all tumor foci require identical local and systemic treatment strategies.

Among these open questions, four are the most immediately actionable for future research: (1) oncological outcomes of breast conservation after neoadjuvant therapy specifically in multicentric disease; (2) optimal radiotherapy boost planning for multiple lumpectomy beds; (3) whether routine preoperative MRI offers a net benefit in this specific subgroup; and (4) the clinical implications of genomic heterogeneity between separate foci for treatment selection.

### Limitations of This Review

This is a narrative rather than a systematic review, and it carries the limitations inherent to that methodology: study selection was based on relevance and quality as judged by the author rather than a predefined, reproducible search protocol, formal risk-of-bias assessment was not performed, and publication or citation bias cannot be excluded. The underlying evidence itself is heterogeneous, with inconsistent definitions of multifocal and multicentric disease, variable imaging strategies, and differing systemic therapy approaches across the studies discussed, which limits how precisely any single figure quoted here should be interpreted.

## 5. Conclusions

The categorical historical position that multicentric breast cancer mandates mastectomy is no longer supported by the totality of the evidence, but it has not been replaced by an equally categorical rule in the other direction, nor should it be. What has emerged instead is a conditional, multidisciplinary pathway: for appropriately selected patients with multicentric disease, breast-conserving surgery appears to provide oncological outcomes that are not inferior to those reported after mastectomy, although direct randomised comparison remains unavailable, provided that complete excision with negative margins is achieved, adjuvant radiotherapy is administered, and an acceptable cosmetic outcome can be attained. This approach depends on adequate, histologically confirmed preoperative imaging, resectable disease amenable to oncoplastic techniques, access to appropriate adjuvant radiotherapy, guideline-concordant systemic therapy, and—where relevant—informed consideration of hereditary cancer risk. Under these conditions, local recurrence rates comparable to those reported in contemporary breast-conservation series of selected unifocal breast cancers can be achieved. The open questions identified here—particularly the necessity of routine MRI, radiotherapy planning for multicentric resection, and outcomes after neoadjuvant therapy—mean that patient selection, informed consent, and multidisciplinary discussion remain as important to outcomes as surgical technique itself. The future of MIBC surgery is unlikely to be defined by a universal preference for conservation or mastectomy, but by accurate disease characterisation, multidisciplinary selection, and matching the extent of surgery to tumor biology rather than anatomical multiplicity alone.

**Figure 2:**
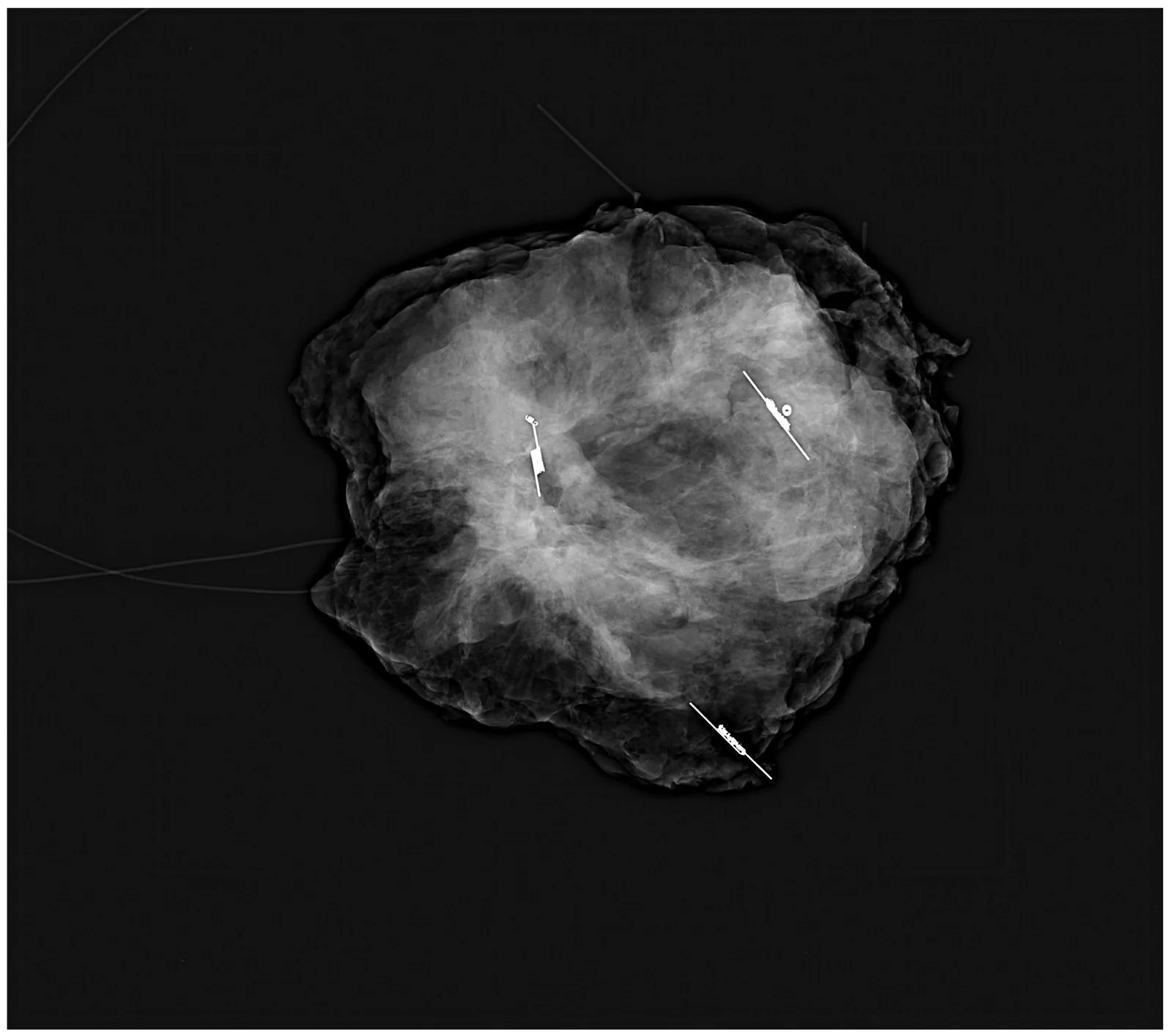
Specimen radiograph from a 48-year-old patient, illustrating successful breast-conserving surgery for multicentric grade 2 invasive lobular carcinoma comprising three tumour foci in the upper outer and lower outer quadrants, spanning 70 mm. Breast conservation was facilitated by preoperative deployment of three SAVI SCOUT^®^ reflectors (Merit Medical Systems, Inc., South Jordan, UT, USA) (one per focus) for radar-guided intraoperative localisation, together with an oncoplastic technique for reconstruction of the resulting defect; final surgical margins were tumour-free.

A representative case illustrating successful multicentric breast conservation is shown in Figure 2.

## Figures and Tables

**Table 1:** Key characteristics of the principal studies underlying the evidence base.

ACOSOG Z11102 (Alliance) [2]	Prospective, Single-Arm	2–3 Biopsy-Proven Ipsilateral Foci, ≤2 Quadrants, No NST Permitted	MRI Initially Mandatory, Later Optional	5 Years (Median)	5-yr Local Recurrence 3.1%
MIPA study [15]	Prospective cohort	Patients undergoing screening or diagnostic breast MRI	MRI (index test)	Not stated	MRI changes the surgical plan more often than mammography/ultrasound
De Lorenzi et al. matched-cohort [16]	Retrospective, matched	100 oncoplastic BCS vs. 100 mastectomy, MC/MF disease	Not specified	94 months (median)	OS/DFS similar between groups; local events non-significantly higher after BCS
Argentinian cohort [17]	Retrospective	Stage I–II MF/MC (n = 91) vs. unifocal (n = 1097)	No routine MRI	Not stated	5-yr locoregional recurrence 3.3% vs. 5.9%
Turkish cohort [18]	Retrospective	246/2155 MF/MC; 68 selected for BCS	Routine MRI	12 months (median)	No local recurrences; 90% negative margins at first surgery
Comparative meta-analysis (Althawadi et al.) [21]	Systematic review and meta-analysis, 17 studies	29,711 patients (9587 BCS, 20,124 mastectomy)	Mixed across studies	62 months (median)	No difference in LR/LRR/DR/DFS; OS advantage lost in upfront-surgery sensitivity analysis

**Table 2:** Oncoplastic technique selection by tumor quadrant and estimated resection volume, for use in preoperative planning of multisite (multifocal/multicentric) resection.

Quadrant/Location	Level I (<20% Volume)	Level II (20–50% Volume)	Volume Replacement (>50% or Minimal Local Reserve)	Key Technical Note
Upper outer quadrant	Local glandular advancement; round-block	Inferior or superomedial pedicle mammaplasty (Wise pattern)	LICAP flap	Most forgiving quadrant; site of majority of tumors
Upper inner/central-retro areolar (“no man’s land”)	Batwing mastopexy (limited utility beyond ~15–20%)	Extended pedicle; matrix-rotation flap	Pedicled pectoralis major myofascial flap	Least mobile tissue; low threshold for referral to a specialist oncoplastic center
Lower pole (6 o’clock)	Local advancement	Superior or superomedial pedicle mammaplasty, Wise or vertical pattern	Rarely required	Tolerates the largest resections with best cosmetic reserve
Lower inner quadrant	Inferior pedicle rotation	V-mammaplasty; superomedial pedicle	Intercostal artery perforator flap	—
Lower outer quadrant	Local advancement	J-mammaplasty; superior or superomedial pedicle	LICAP/TDAP flap	—
Central/subareolar (NAC involved)	Round-block, peri-areolar de-epithelialization	Wise-pattern with planned NAC reconstruction if excised	Pedicled pectoralis major myofascial flap	NAC preservation feasibility assessed separately for each focus

**Table 3:** Current guideline positions on breast-conserving surgery in multicentric disease.

Guideline Body	Position On BCS In Multicentric Disease	Key Caveat
American Society of Breast Surgeons (ASBrS)	Multicentric disease not amenable to oncoplastic surgery remains listed as an absolute contraindication, but oncoplastic technique and neoadjuvant therapy are explicitly recognized as expanding eligibility, and practice is expected to adapt to patient preference and evolving evidence [13].	Caveat applies only where oncoplastic surgery cannot achieve the goal—not a blanket rule against conservation.
NCCN	No disease-specific rule for multicentricity; general early breast cancer framework applies, with the surgical decision left to individualized, multidisciplinary judgement.	No disease-specific caveat—defers entirely to multidisciplinary judgement.
ESMO	Similarly, does not issue a prescriptive rule; emphasizes multidisciplinary team (MDT) selection within its general early breast cancer framework.	No disease-specific caveat—no prescriptive criteria issued for MIBC.
AGO (German Cancer Society, Breast Committee)	Permits breast-conserving therapy in multifocal/multicentric disease conditional on R0 resection at every focus but explicitly notes multicentricity may still justify mastectomy even after a good neoadjuvant response [31,32].	Caution applies even after a good neoadjuvant response, reflecting limited long-term local control data in that specific setting.

**Table 4:** Minimum requirements before offering breast-conserving surgery in multicentric disease.

Domain	Minimum Requirement Before Offering BCS
Imaging	Disease extent defined by preoperative imaging; any suspicious MRI lesion confirmed histologically before it changes the surgical plan
Biology	Guideline-concordant systemic therapy plan established according to biomarker status (endocrine, HER2-targeted, or chemotherapy)
Surgery	All identified foci excisable to a negative margin (“no ink on tumour” for every invasive focus and any DCIS coexisting with invasive disease at that focus; 2 mm applies only to a purely in situ, DCIS-only focus)
Reconstruction	Access to oncoplastic expertise for volume-displacement or volume-replacement technique where multicentric resection is planned
Radiation	Whole-breast radiotherapy with a boost to each lumpectomy bed is feasible
Patient factors	Informed preference reached after multidisciplinary discussion, including hereditary risk where relevant

## Data Availability

No new data were created or analyzed in this study. Data sharing is not applicable to this article.
